# The Efficacy of Combined Medication With Methylprednisolone and Erythropoietin in the Treatment of Ischemia-Reperfusion Injury to the Spinal Cord in Patients With Cervical Spondylotic Myelopathy

**DOI:** 10.7759/cureus.14018

**Published:** 2021-03-21

**Authors:** Fahri Eryilmaz, Umar Farooque

**Affiliations:** 1 Neurological Surgery, Hitit University Erol Olcok Training and Research Hospital, Corum, TUR; 2 Neurology, Dow University of Health Sciences, Karachi, PAK

**Keywords:** spinal cord, erythropoietin, methylprednisolone, myelopathy, ischemia-reperfusion injury, cervical spondylotic myelopathy, efficacy, combined medication

## Abstract

Introduction

Cervical myelopathy (CM) is caused by degenerative or congenital changes in the discs and soft tissues of the cervical spine, leading to chronic compression of the spinal cord. The current treatment for moderate-to-severe CM is surgical decompression, which is effective in most cases; however, it can cause inflammation of the nervous system and spinal cord reperfusion injury, resulting in perioperative neurological complications and suboptimal neurological recovery. The aim of this study was to investigate the therapeutic effects of the combination of erythropoietin and methylprednisolone in the treatment of ischemia-reperfusion injury to the spinal cord and to analyze its effects on the levels of interleukin-1 beta (IL-1β), interleukin-1 receptor antagonist (IL-1RA), and interleukin-8 (IL-8).

Materials and methods

This study included 110 patients admitted to the hospital due to cervical spondylotic myelopathy. They were randomized into two groups of 55 patients each: a control and an observation group. In both groups of patients, fusion internal fixation and anterior cervical discectomy were performed. The difference, however, was that the control group received a rapid intravenous injection of 30 mg/kg methylprednisolone 30 minutes prior to spinal cord decompression, while the observation group received an intravenous injection of 30 mg/kg methylprednisolone and 3,000 U/kg erythropoietin 30 minutes before spinal cord decompression. The study was approved by the Hospital Ethical Committee of the Dow University of Health Sciences, Karachi. The neurological function of both groups of patients was assessed before the procedure and three months after the treatment using the Japanese Orthopedic Association (JOA) method of assessing spinal cord function (40-point rating method). Enzyme-linked immunosorbent assay (ELISA) was performed to measure the levels of neuron-specific enolase (NSE), S-100β, IL-1RA, IL-1β, and IL-8 in both groups. The quality of life of patients in both groups was assessed three months after the treatment with the World Health Organization Quality of Life assessment instrument (WHOQOL-100).

Results

Before the treatment, there was no significant variance between the two groups in the JOA score and the 40-point rating method. Similarly, there was no significant difference in the levels of IL-1β, IL-1RA, and IL-8 between the two groups (p-value = 0.262, 0.387, and 0.154 respectively) prior to the treatment. Three months after the treatment, the levels of IL-1β and IL-8 in the observation group were 21.83 ±3.65 ng/l and 357.07 ±32.36 ng/l respectively, both lower than the control group value (p-value = 0.026, 0.028 respectively). The level of IL-1RA in follow-up was 21.59 ±1.15 ng/l, which was higher than that in the control group. Three months after the treatment, all the WHOQOL-100 parameters of the observation group for psychology, physiology, social relations, independence, spirituality, environment, and general quality of life were higher than those of the control group; the variance among the groups was statistically significant (p-value: <0.001).

Conclusions

The combination therapy with erythropoietin and methylprednisolone is effective for ischemia-reperfusion injuries of the spinal cord. It also reduces S-100β and NSE, inhibits IL-1β, and increases IL-8 and IL-1RA. Therefore, it preserves and improves spinal nerve function and the quality of life of patients.

## Introduction

Cervical myelopathy (CM) refers to the compression of the cervical spinal cord due to either a disc herniation or cervical spinal stenosis [1]. The pathogenesis of myelopathy of the cervical spine is a degenerative pathological change in the cervical vertebrae [2,3]. Due to the appearance of soft tissue outside the spinal cord and the compression of the peripheral bone, the function of the spinal nerve deteriorates, and the anatomical structure changes. Cervical spine myelopathy can lead to disorders of the blood supply to the spinal cord and venous reflux, cause myelomalacia and bone marrow necrosis, and lead to spinal nerve malfunction [4,5]. Although surgery may be effective in alleviating the spinal cord compression, it may also cause some degree of ischemia-reperfusion injury, thereby making it difficult to restore the nerve function after decompression [6,7].

A reperfusion injury from spinal cord ischemia means that once the blood supply to the spinal cord is restored, nerve function cannot be improved or may even get worsened [8]. In some extremely severe cases, the spinal nerve cloud can cause delayed irreversible neuronal death. Methylprednisolone is an effective drug in the treatment of spinal ischemia-reperfusion. However, it has been observed that high doses of methylprednisolone can cause complications such as gastrointestinal bleeding and bone necrosis [9,10]. Erythropoietin largely determines the oxygenation state of tissues. Erythropoietin is highly expressed in central nervous system cells, exerting neurotrophic, neuronal anti-apoptotic, antioxidant, and anti-inflammatory effects through an autocrine and paracrine approach. There are currently few studies on the therapeutic effects of the combination of erythropoietin and methylprednisolone in the treatment of ischemic and reperfusion spinal cord injuries in patients with CM [11,12]. Against this background, this study analyzed the therapeutic effects of combined erythropoietin and methylprednisolone therapy on the treatment of ischemia-reperfusion injuries of the spinal cord in patients with cervical spine myelopathy and its impact on the levels of interleukin-1 beta (IL-1β), interleukin-1 receptor antagonist (IL-1RA), and interleukin-8 (IL-8), which are considered to be the indicators of ischemia-reperfusion injury to the spinal cord in patients with cervical spondylotic myelopathy. The primary aim of the study was to observe the mean rise in the Japanese Orthopedic Association (JOA) score, S-100β levels, and neuron-specific enolase (NSE) levels among the two groups of patients [those who received methylprednisolone alone (control) and those who received the combined therapy of erythropoietin and methylprednisolone (observation)] three months after the treatment. The secondary aim was to measure the levels of IL-1β, IL-1RA, and IL-8, and analyze the World Health Organization Quality of Life assessment instrument (WHOQOL-100) scores three months after the treatment.

## Materials and methods

This study was conducted at the Dow University of Health Sciences, Karachi for a duration of six months from March 2020 to August 2020. It was approved by the Hospital Ethical Committee of the Dow University of Health Sciences. It included 110 patients admitted to our hospital for the treatment of cervical spondylotic myelopathy. They were randomized into two groups of 55 patients each as a control and an observation group. The inclusion criteria were as follows: patients meeting the diagnostic standards for cervical spondylotic myelopathy and under the age of 75 years, with an MRI degeneration of a single section or two adjacent sections of the lumbar disc, which is an important sign of vascular myelopathy, without peripheral vascular diseases, which affect the function of the upper limbs, and having no diseases of the heart, liver, kidneys, and lungs. All selected patients were informed about the nature of the study and informed consent was obtained.

Patients who refused to participate in the study, those who had a mental illness, those who were ≥75 years of age, and those who had a heart or kidney disease were excluded from the study.

A total of 110 patients were randomly assigned to a control group and an observation group of 55 patients each. A comparative analysis of the two groups pertaining to gender, age, weight, height, course of the disease, and site of injury (one or two adjacent segments) is presented in Table 1.

**Table 1:** Comparison of general data between the two groups

Groups	Number of patients	Gender (male/female)	Average age, years	Mean body weight, kg	Mean height, cm	Disease course, years	Site of the lesion (single section/two adjacent sections)
Observation group	55	29/26	57.53 ±3.39	66.17 ±3.82	166.24 ±9.37	1.88 ±0.41	32/23
Control group	55	28/27	57.45 ±2.96	66.21 ±3.73	165.18 ±8.75	1.85 ±0.52	31/24
t/X^2^		0.773	0.464	0.781	1.105	0.706	0.999
P-value		0.131	0.149	0.116	0.338	0.254	0.097

Table 1 shows the demographic features of the patients. The average age in the observation group was 57.53 ±3.39 years, and that in the control group was 57.45 ±2.96 years; the mean body weight was 66.17 ±3.82 kg and 66.21 ±3.73 kg respectively. The disease duration was 1.88 ±0.41 years and 1.85 ±0.52 years in the observation and control groups respectively. In both groups of patients, fusion internal fixation and anterior discectomy for two adjacent sections or single section intervertebral disk were performed. The operation was performed in both groups by the same group of surgeons. Thirty minutes before spinal cord decompression, the control group was given a 30 mg/kg intravenous injection of methylprednisolone (completed in 15 minutes), while the observation group was given a 30 mg/kg intravenous injection of methylprednisolone and 3,000 U/kg erythropoietin (completed in 15 minutes).

After the surgery, each patient in both groups was orally administered 80 mg methylprednisolone once daily for two consecutive weeks. The neurological function of both groups of patients before the procedure and three months after the treatment was assessed using the JOA scale and the method of spinal cord function classification. The greater the score, the better the state of neurological function. A biochemical enzyme-linked immunosorbent assay (ELISA) test was performed to measure the levels of S-100β, NSE, IL-1β, IL-1RA, and IL-8 in the two groups before the procedure and three months after the treatment. The patient quality of life in both groups was assessed three months after the treatment with WHOQOL-100. WHOQOL-100 covers six dimensions related to the quality of life, namely physiology, psychology, independence, social relations, environment, and spirituality. In addition, WHOQOL-100 includes 24 items, and each item contains four questions plus four additional questions on general health and overall quality of life: WHOQOL-100 includes 100 questions in total. The greater the score in each dimension, the greater the quality of life. Statistical analysis was performed using SPSS Statistics 22.0 (IBM, Armonk, NY). Measurement data were presented as mean ±standard error (x ±s) and were subjected to an independent t-test. The calculated data were subjected to the chi-square test. A p-value of <0.05 was considered to represent a statistically significant difference.

## Results

Before the treatment, there was no significant difference between the two groups in the JOA score and the 40-point rating method, but those increased significantly in the observation group three months after the treatment, as shown in Table 2.

**Table 2:** Comparison of neurological function score between the two groups (x ±s) x ±s: mean value ±standard error; JOA: Japanese Orthopedic Association

Groups	Number of patients	JOA score		40-point rating method	
		Before the treatment	Three months after the treatment	Before the treatment	Three months after the treatment
Observation group	55	10.25 ±1.72	18.43 ±2.81	31.04 ±3.35	39.22 ±4.07
Control group	55	10.31 ±2.05	15.06 ±2.93	30.97 ±3.11	35.66 ±4.13
t		0.204	5.337	0.403	6.018
P-value		0.438	0.035	0.326	0.029

The JOA score in the observation group before the treatment was 10.25 ±1.72 and that in the control group was 10.31 ±2.05. Three months after the treatment, it was 18.43 ±2.81 and 15.06 ±2.93 respectively. The 40-point rating method score before the treatment in the observation group was 31.04 ±3.35 and that in the control group was 30.97. Three months after the treatment, it was 39.22 ±4.07 and 35.66 ±4.13 respectively.

Before the treatment, there was no significant variance in S-100β and NSE levels between the two groups. Three months after the treatment, the levels of S-100β and NSE in the observation group were significantly higher than the corresponding values in the control group, as shown in Table 3. The S-100β levels three months after treatment were 0.18 ±0.12 in the observation group and 0.22 ±0.14 in the control group. The NSE levels three months after the treatment were 9.61 ±0.65 and 12.13 ±0.96 respectively in the observation and control groups.

**Table 3:** Comparison of S-100β and NSE levels between the two groups (x ±s; g/l) x ±s: mean value ±standard error; NSE: neuron-specific enolase

Groups	Number of patients	S-100β		NSE	
		Before the treatment	Three months after the treatment	Before the treatment	Three months after the treatment
Observation group	55	0.29 ±0.22	0.18 ±0.12	15.72 ±1.43	9.61 ±0.65
Control group	55	0.28 ±0.26	0.22 ±0.14	15.64 ±1.57	12.13 ±0.96
t		0.206	5.092	0.314	6.027
P-value		0.562	0.041	0.421	0.032

Before the treatment, there was no significant difference in IL-1β, IL-1RA, and IL-8 between the two groups (p-value = 0.262, 0.387, and 0.154 respectively). Three months after the treatment, the levels of IL-8 and IL-1β in the observation group were 21.83 ±3.65 ng/l and 357.07 ±32.36 ng/l respectively, both lower than the control group values (p-value = 0.026, 0.028 respectively). The level of IL-1RA during the follow-up in the observation group was 21.59 ±1.15 ng/l, which was higher than that in the control group, as shown in Table 4.

**Table 4:** Comparison of the levels of IL-1β, IL-1RA, and IL-8 between the two groups (x ±s; ng/l) x ±s: mean value ±standard error; IL-1β: interleukin-1 beta; IL-R: interleukin-1 receptor antagonist; IL-8: interleukin-8

Groups	Number of patients	IL-1β		IL-1RA		IL-8	
		Before the treatment	Three months after the treatment	Before the treatment	Three months after the treatment	Before the treatment	Three months after the treatment
Observation group	55	45.35 ±4.26	21.83 ±3.65	16.27 ±2.12	21.73 ±1.24	843.76 ±57.92	357.07 ±32.36
Control group	55	45.18 ±3.84	31.04 ±2.28	16.14 ±2.07	21.59 ±1.15	841.35 ±60.27	417.88 ±41.43
t		0.654	8.054	0.132	6.715	0.708	6.926
P-value		0.262	0.026	0.387	0.031	0.154	0.028

Three months after the treatment, all the WHOQOL-100 results of the observation group for psychology, physiology, social relations, independence, spirituality, environment, and overall quality of life were greater than that of the control group; the variance among the groups was statistically significant, as shown in Table 5 (p-value: <0.001).

**Table 5:** Comparison of the quality of life parameters between the two groups three months after the treatment (x ±s) x ±s: mean value ±standard error

Groups	Number of patients	Physiology	Psychology	Social relations	Independence	Environment	Spirituality	Overall quality of life
Observation group	55	73.36 ±7.95	81.84 ±11.91	77.23 ±8.54	87.29 ±12.65	83.21 ±8.82	83.51 ±10.88	68.19 ±7.62
Control group	55	67.44 ±9.13	81.25 ±11.06	69.12 ±6.45	79.24 ±11.38	71.75 ±9.13	75.46 ±7.71	59.53 ±6.51
t		10.645	12.156	13.903	17.105	19.424	18.016	17.182
P-value		<0.001	<0.001	<0.001	<0.001	<0.001	<0.001	<0.001

## Discussion

Cervical spine myelopathy is a common clinical condition. Surgical decompression has long been recognized as the dominant treatment method for this disease, and it involves relieving pressure on the spinal cord and maintaining spine stability [13,14]. However, since the compressed spine already exhibits anaerobic changes, the sudden increase in blood flow following surgical decompression may result in ischemia-reperfusion injury [15]. Currently, there is no specific treatment for ischemia-reperfusion injuries of the spine. Methylprednisolone is a synthetic glucocorticoid that has a therapeutic effect on ischemia-reperfusion injuries of the spinal cord due to its role in inhibiting lipid peroxidase and the post-traumatic inflammatory response. This drug has a strong anti-inflammatory effect and is considered the standard medication for acute spinal cord injury [16,17]. Relevant literature indicates that a high dose of methylprednisolone can cause a variety of side effects that are not beneficial for the improvement of neurological function. Research has shown that erythropoietin and its receptors are widely expressed in organs and tissues of the human body, which have a potential for cytoprotection [18]. Erythropoietin has a neurotrophic effect that may increase the tolerance of brain tissue to ischemia-hypoxia and exerts neuroprotective effects by counteracting cell apoptotic, antioxidant, and anti-inflammatory effects [19,20]. This study showed that three months after the treatment, the JOA scores and the 40-point grading method in the observation group, who were administered methylprednisolone in combination with erythropoietin, were significantly higher than in the control group, who were given methylprednisolone alone (p-value = 0.025, 0.019 respectively) This indicates that the combined therapy with erythropoietin and methylprednisolone may promote the improvement of neurological functions in patients with cervical spine myelopathy.

Studies have shown that IL-1β is involved in the early course of ischemia-reperfusion injury to the spinal cord. Increased IL-1β expression following reperfusion injury is an important molecular basis for damage to the bloodstream barrier of the spinal cord [19-21]. Studies show that IL-1RA can compete with the IL-1 receptor to block the biological effects of IL-1, thereby defending the pathological damage caused by IL-1β and ameliorating ischemic damage. The expression or positive production of IL-8 will not occur in normal spinal cord tissue, but only in the process of ischemia-reperfusion injury (especially around endothelial cells). IL-8 is mainly derived from endothelial cells [22]. Kwiecien et al. investigated the role of antithrombin in reducing inflammation induced by reperfusion ischemia and ameliorating spinal cord injury in rats and found that antithrombin can significantly inhibit IL-8 expression [23,24]. This study showed that three months after the treatment, the levels of IL-1β and IL-8 in the observation group were lower than in the control group, while the level of IL-1RA in the observation group was higher than in the control group (p-value = 0.021). This indicates that the combination therapy of erythropoietin and methylprednisolone has advantages in inhibiting IL-1 and IL-8 and promoting IL-1RA, thereby ensuring better preservation of spinal nerve function. Moreover, these studies show that the combination of erythropoietin and methylprednisolone can significantly improve the quality of life of patients compared to therapy involving methylprednisolone alone [25,26].

## Conclusions

Based on our findings, the combined treatment with erythropoietin and methylprednisolone resulted in a significant therapeutic effect on the treatment of ischemia-reperfusion injuries of the spinal cord. This combination therapy also played an effective role in reducing S-100β and NSE, inhibiting IL-1β, and increasing IL-8 and IL-1RA, thereby preserving and improving spinal nerve function and patients' quality of life. The combined treatment with erythropoietin and methylprednisolone deserves more support and endorsement to be implemented in the hospitals.
